# 397. Impact of School Opening Model on Cases of SARS-CoV-2 in Surrounding Communities: A Nationwide, Retrospective Cohort Study

**DOI:** 10.1093/ofid/ofab466.598

**Published:** 2021-12-04

**Authors:** Westyn Branch-Elliman, Zeynep Ertem, Elissa Perkins, Polly van den Berg, Isabella Epshtein, Nathorn Chaiyakunapruk, Fernando Wilson, Emily Oster, Richard Nelson

**Affiliations:** 1 Veterans Affairs Boston Center for Healthcare Organization and Implementation Research, Boston, Massachusetts; 2 SUNY Binghamton, Binghamton, New York; 3 Boston University, Boston, Massachusetts; 4 BIDMC, Boston, Massachusetts; 5 VA Boston CHOIR, Boston, Massachusetts; 6 University of Utah, Salt Lake City, Utah; 7 Brown University, Providence, Puerto Rico; 8 IDEAS Center, VA Salt Lake City Healthcare System; 9 Division of Epidemiology, University of Utah School of Medicine, Salt Lake City, UT

## Abstract

**Background:**

Early in the COVID-19 pandemic, elementary and secondary schools were closed. There was variation in school opening mode (traditional, hybrid, remote) in fall 2020.The aim of this national, retrospective cohort study is to evaluate the impact of in-person learning on community incidence of SARS-CoV-2 and COVID-19-related deaths.

**Methods:**

Data were extracted from several data sources. School opening mode was collected from the Burbio school tracker, which tracks school openings in a sample of school districts across the US. Incidence of SARS-CoV-2 and COVID-19 related deaths were obtained from the CDC. Data on community-level SARS-CoV-2 mitigation measures were obtained from the Oxford University COVID-19 Government Response Tracker. The effect of school mode on SARS-CoV-2 cases and deaths/100,000 during the 12-weeks following the start of school was estimated using a log-linear model with state, week, and state-week fixed effects. Models were stratified by 9 US Census divisions and adjusted for variables determined a priori to be potentially associated with the outcome.

**Results:**

519 US counties were included (Figure 1); mean cases of COVID-19 were increasing across all regions during the weeks following the start of school, regardless of school mode. Adjusted absolute differences in COVID-19 cases in counties with hybrid and traditional school opening modes relative to fully remote learning models are presented in Figure 2. In the Northeast and Midwest regions of the country, COVID-19 case rates were not statistically different between different school modes. However, in the South and West regions, there was a statistically significant increase in cases per week among counties that opened in an in-person relative to remote learning model, ranging from 17.1 (95% CI: 0.3-33.8) to 24.4 (95% CI: 7.3-41.5) in the South and from 19.0 (95% CI: 8.8-29.3) to 109.2 (95% CI: 50.4-168.0) in the West. There was no impact of school opening mode on COVID-19-related deaths.

Figure 1. Map with distribution of counties and school opening mode across the United States

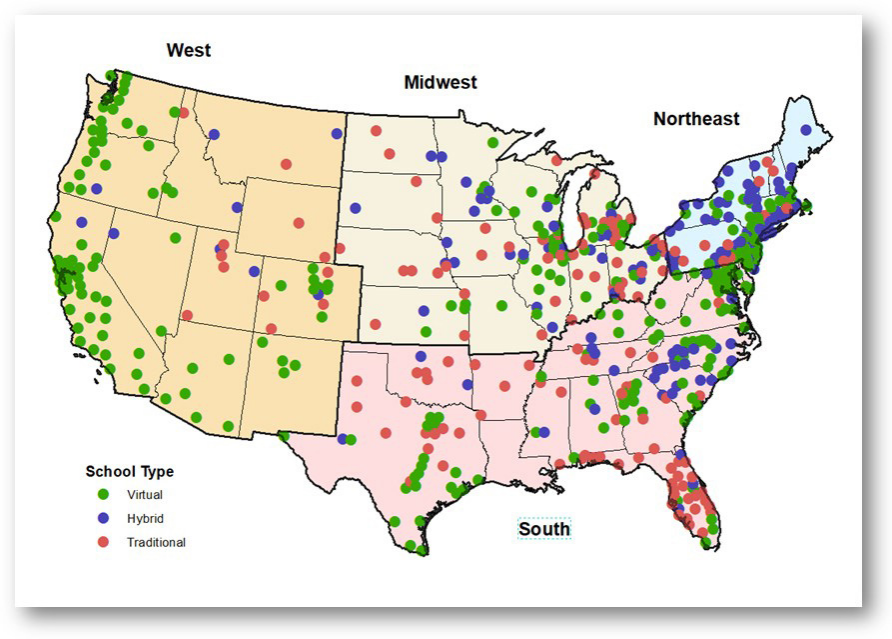

Figure 2. Impact of school opening mode on subsequent cases of SARS-CoV-2, stratified by region.

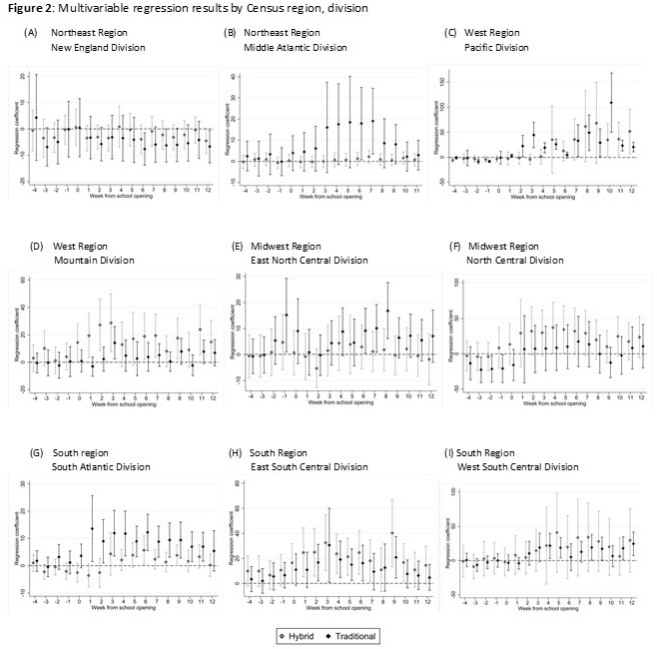

**Conclusion:**

Impact of school mode on community case-rates of SARS-CoV-2 varied across the US. In some areas of the country, traditional school mode was associated with increases in case rates relative to virtual while there were no differences in other regions.

**Disclosures:**

**All Authors**: No reported disclosures

